# Results of arthroscopic suspension fixation for acute posterior cruciate ligament avulsion fractures in adolescents: a retrospective cohort study

**DOI:** 10.1097/JS9.0000000000002980

**Published:** 2025-07-09

**Authors:** Congliang Chen, Song Wang, Jinlong Tang, Tianle Liu, Zhengya Zhu, Wei Zheng

**Affiliations:** a Department of Orthopaedic Surgery, Sports Medicine, The Affiliated Hospital of Xuzhou Medical University, Xuzhou, China; b The First Clinical College, XuZhou Medical University, Xuzhou, China

**Keywords:** adolescents, arthroscopic, epiphyseal line, posterior cruciate ligament avulsion fractures, sports medicine

## Abstract

**Background:**

This study aimed to evaluate the clinical efficacy of arthroscopic suspension fixation in the treatment of acute posterior cruciate ligament (PCL) avulsion fractures in adolescents.

**Materials and methods:**

We retrospectively analyzed the clinical data of adolescent patients with PCL tibial avulsion fractures who underwent arthroscopic suspension fixation between May 2013 and November 2022. All the patients underwent arthroscopic suspension fixation. Clinical and functional outcomes were evaluated using the Lysholm score, the 2000 International Knee Documentation Committee (IKDC) subjective score, and the IKDC examination form. The follow-up period for all patients was at least 2 years, with an average of 29.8 months. At the final follow-up, differences in leg length between the lower limbs were measured and assessed.

**Results:**

All patients met the study criteria and completed at least 2 years of follow-up, averaging 29.8 months. The mean age was 15.4 years (range, 12–18 years). The mean Lysholm score increased from 21.55 ± 7.18 preoperatively to 95.25 ± 2.17. The mean 2000 IKDC subjective score improved from 18.25 ± 5.49 to 94.9 ± 2.43, and knee range of motion improved from 39.25° ± 6.71° to 136.5° ± 4.12°. The IKDC examination grade also improved significantly, with no bilateral limb length differences exceeding 1 cm at the final follow-up, and no patient reported any noticeable limb length discrepancies.

**Conclusion:**

This suspensory arthroscopic technique is a minimally invasive and safe treatment for PCL tibial avulsion fractures in adolescents. Suspensory fixation results in satisfactory outcomes and achieves effective knee stability and fracture union.

**Level of evidence:**

IV, therapeutic study.

## Introduction

The posterior cruciate ligament (PCL) is a crucial stabilizing structure of the knee joint that plays an essential role in maintaining rotational and posterior stability during movement^[1]^. PCL tibial avulsion fractures are a specific type of PCL injury. Although these fractures are relatively rare worldwide^[2]^, studies in Western countries have suggested that they account for approximately 20% of knee ligament injuries^[3]^. However, in Asia, its incidence is expected to increase because of the growing popularity of electric vehicles^[4]^.

This type of injury is exceptionally rare in children and adolescents. Research on the epidemiology, treatment methods, and long-term outcomes of PCL tibial avulsion fractures in these age groups is limited. Additionally, the current literature is primarily limited to case reports and small case series^[5]^.

PCL injuries are typically caused by high-energy trauma^[6]^. In skeletally immature patients, the developing bone tissue and growth plates are more vulnerable than the ligamentous structures^[7]^. This unique physiological structure and associated pathogenic factors may explain why adolescent patients are prone to PCL tibial avulsion fractures. Improper treatment of PCL avulsion fractures can lead to posterior knee instability, pain, and other symptoms that impair knee stability^[8]^. Persistent instability can alter the stress distribution around the joint, whereas normal mechanical stress plays a vital role in the development of growth plates^[9]^, so that improper treatment can significantly disrupt the growth and development of adolescents.

Treatment options for such fractures include conservative management, open surgery, and minimally invasive arthroscopic surgery^[6]^. However, there is no consensus regarding the most appropriate treatment for adolescents. Conservative management remains controversial because of the variable outcomes; some patients recover significantly, whereas others do not. Notably, conservative treatment carries risks, such as persistent ligament insufficiency, secondary displacement, and early onset osteoarthritis, leading to joint degeneration or meniscal injuries. Both open surgery and arthroscopic techniques have yielded promising results. Although traditional open surgery can effectively treat avulsion fractures, it generally requires a larger incision, leading to considerable scarring that can negatively affect postoperative joint function. Furthermore, a larger incision increases the risk of infection and potential neurovascular injury^[10]^.

The hardware used in open reduction included screws^[11]^, sutures^[12]^, anchors^[13]^, screw and washer^[14]^. In arthroscopic procedures, common hardware includes sutures, screws, and staples^[6]^. Currently, most surgeons favor sutures for the arthroscopic treatment of PCL tibial avulsion fractures in adolescents. However, no clinical studies have evaluated the outcomes of suspension fixation devices in these cases.

This study presents a modified arthroscopic technique using the Endobutton device for the fixation of PCL tibial insertion avulsion fractures. This method is widely used in anterior cruciate ligament reconstruction^[15]^, acromioclavicular joint separation^[16]^, and distal biceps tendon rupture repair^[17]^. Biomechanical studies suggested that Endobutton provides mechanical stability comparable to direct screw fixation^[18]^. This study aimed to evaluate the clinical efficacy of arthroscopic suspension fixation for the treatment of acute PCL avulsion fractures in adolescents. We hypothesized that this technique would achieve positive clinical and radiographic outcomes and demonstrate its feasibility in clinical practice. This cohort study has been reported in line with the STROCSS guidelines^[19]^.

## Methods

This retrospective cohort study focused on adolescent patients with PCL tibial avulsion fractures who underwent arthroscopic suspension fixation between May 2013 and November 2022. During this period, all eligible patients were consecutively enrolled based on their visit sequence to ensure consistency in patient selection and minimize potential bias.

The inclusion criteria were strictly defined as follows: (1) age under 18 years with an open growth plate; (2) acute injury (with the interval between injury and surgery being less than 3 weeks); (3) PCL tibial avulsion fracture, with displacement greater than 3 mm, confirmed through knee joint X-rays (anteroposterior and lateral views), computed tomography (CT) three-dimensional reconstruction, and magnetic resonance imaging (MRI); (4) treatment with arthroscopic suspension fixation; (5) all patients and their families consented to and were able to complete both the surgical treatment and follow-up. Exclusion criteria included: (1) closed growth plates; (2) significant leg length discrepancy before surgery; (3) concomitant injury to the collateral ligaments, anterior cruciate ligament, or PCL body; (4) patients with severe personality disorders who were unable to complete follow-up, as defined by the Diagnostic and Statistical Manual of Mental Disorders, 5th edition criteria, and confirmed through clinical psychological assessments such as the Minnesota Multiphasic Personality Inventory-2, etc.; (5) associated femoral or tibial plateau fractures; (6) congenital limb deformities or prior knee surgery on the affected side; (7) severe systemic diseases or other medical conditions that could affect surgical outcomes or postoperative recovery. All surgeries were performed by the same senior orthopedic surgeon with >10 years of experience in knee arthroscopy.

**HIGHLIGHTS**

- The incidence of posterior cruciate ligament (PCL) avulsion fractures is relatively low. In adolescents, PCL avulsion fractures have only been reported in isolated case studies in the literature. To our knowledge, no comprehensive retrospective analysis exists in this area. For the first time, our study provides a comprehensive evaluation of the efficacy, safety, and reliability of a suspension fixation technique for treating PCL tibial avulsion fractures in adolescents.
- We have innovated an arthroscopic suspension fixation technique combined with suture fixation, designed to treat PCL avulsion fractures of all types, including comminuted fractures. This technique is suitable for all types of fractures, including comminuted fractures. It achieves high fixation strength while reducing unit-area pressure, thereby minimizing the risk of iatrogenic fractures.
- We conducted postoperative follow-ups on adolescent patients for up to 2 years. The results demonstrate that our method exerts minimal impact on the growth plates of adolescents. No long-term complications, such as limb length discrepancies, were observed. These findings demonstrate that our technique is safe, effective, and reliable for adolescent patients.
- Compared to traditional rigid fixation methods such as screw fixation, elastic fixation offers a significant advantage: it aligns with Frost’s theory of mechanical regulation, which highlights the role of controlled micromovement in enhancing fracture healing.
- This suspension fixation technique is straightforward and highly practical, performed as a minimally invasive procedure under arthroscopic guidance. It has excellent potential for widespread clinical adoption. Moreover, patients can commence functional rehabilitation early, with a low incidence of joint stiffness. The risk of postoperative fracture displacement is minimal, and the fixation strength is sufficient to support early functional recovery.

**Figure 1.**
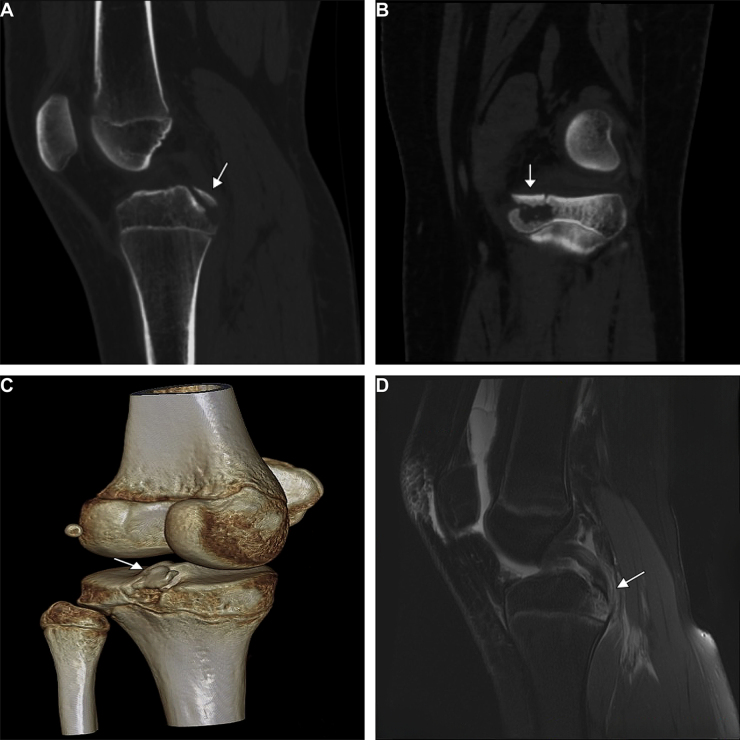
Preoperative imaging evaluation of the patient. (A) Sagittal computed tomography (CT) scan. (B) Coronal CT scan. (C) Three-dimensional CT scan. (D) Sagittal magnetic resonance imaging (MRI). Arrows highlight the avulsion fracture located at the tibial insertion of the posterior cruciate ligament (PCL).

The preoperative evaluation included X-ray, CT, and MRI to confirm the avulsion fracture (Fig. 1) and formulate the preoperative plan. To minimize evaluation bias, the Lysholm score, IKDC subjective score, knee range of motion, and IKDC examination (7 clinical and imaging domains) were independently assessed and recorded by three experienced orthopedic surgeons, with any discrepancies resolved by consensus. This study adhered to the STROCSS guidelines to ensure transparency in the reporting of methods for retrospective cohort studies while minimizing selection bias through standardized patient enrollment, data collection, and outcome evaluation processes^[19]^.

### Surgical technique

**Figure 2.**
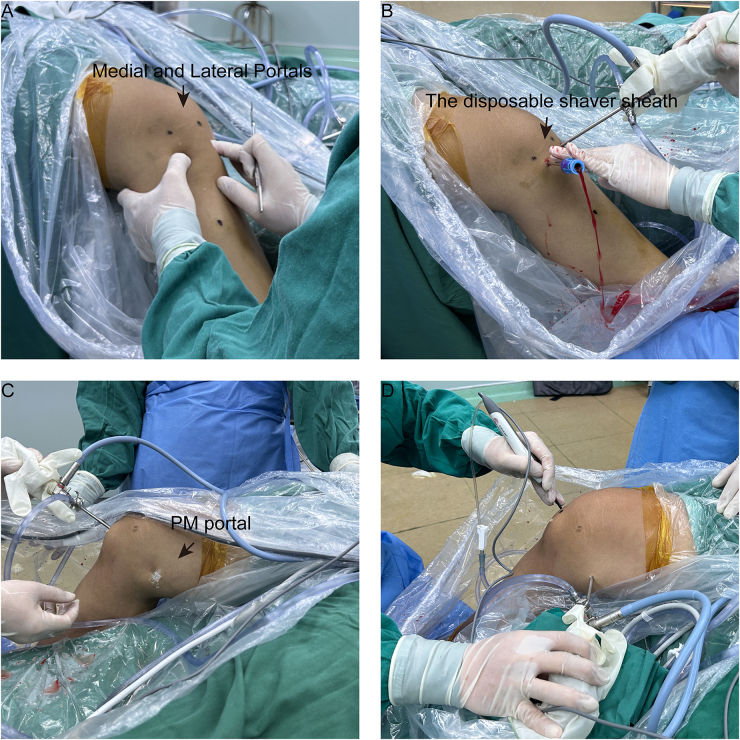
External procedural illustrations. (A) Formation of medial and lateral portals on the patellar ligament for surgical access. (B) Evacuation of intra-articular hematoma to ensure a clear surgical field. (C) Accurate creation of the posteromedial portal using a 16-gauge spinal needle for precise entry. (D) Through the additional posteromedial portal, a disposable wand and shaver were used to clear the hematoma and soft tissue around the fracture site. PM, posteromedial.

**Figure 3.**
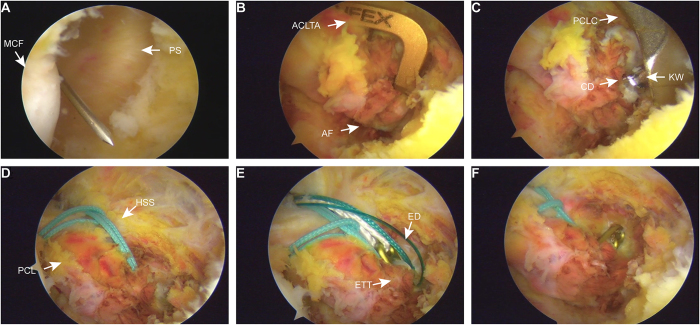
Arthroscopic operation images. (A) A posteromedial portal was carefully created with a 16-gauge spinal needle, observed through the anterolateral portal. (B) An ACL tibial guide was utilized for precise alignment of the avulsed fragment. (C) A tibial tunnel was drilled under the guidance of a K-wire, with the PCL protector. (D) High-strength sutures were looped around the PCL for secure fixation. (E) The Endobutton device was placed atop the avulsed fragment. (F) The fragment was stably secured using high-strength sutures and the Endobutton fixation device. MCF: medial condyle of femur, PS: posterior septum, ACLTA: anterior posterior cruciate ligament tibial aimer, AF: avulsion fracture, PCLC: PCL catcher, CD: cannulated drill, KW: Kirschner wire, HSS: high-strength suture, PCL: posterior cruciate ligament, ETT: exit of tibial tunnel, ED: Endobutton device.

Routine intravenous antibiotics were administered pre-operatively to prevent perioperative infections. Once general anesthesia was successfully administered, patients were positioned supine. After a thorough physical examination, a tourniquet was tied to the root of the affected thigh, and the gap between the tourniquet and the skin was closed with an antimicrobial membrane. Following antiseptic preparation and draping, two 0.6 cm incisions were made approximately 0.8 cm from either side of the patellar tendon at the level of the knee joint space to access the joint cavity (Fig. 2A). Given that recent fractures often cause significant intra-articular bleeding, a disposable shaver sheath (Smith & Nephew, USA) was inserted to fully evacuate the hematoma and ensure a clear view during the procedure (Fig. 2B). Following diagnostic arthroscopy, the fatty tissue between the anterior and posterior cruciate ligaments was excised until the posterior capsule was clearly visible. An additional posteromedial portal was established during the arthroscopy (Figs 2C and 3A).

**Figure 4.**
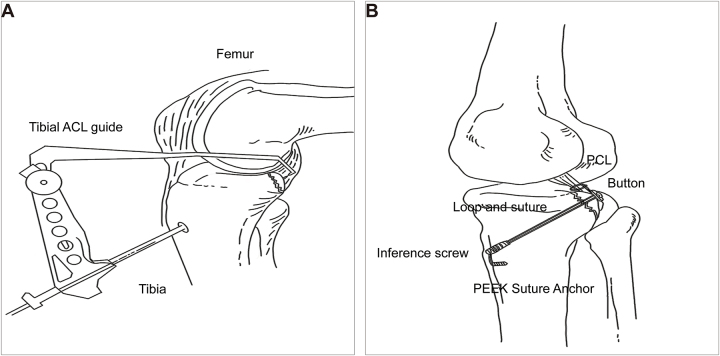
Schematic representation of surgical techniques. (A) The avulsed fragment’s center was precisely located using an ACL tibial guide, followed by controlled drilling of a K-wire. (B) A combination of high-strength sutures and the Endobutton device achieved robust dual fixation, further reinforced by double fixation at the anterior portion of the tibial tunnel.

Through this portal (Fig. 2D), a disposable wand and shaver were used to clear the hematoma and soft tissue around the fracture site, refreshing the fracture. The arthroscope was then repositioned in the posteromedial portal and an atibial anterior cruciate ligament (ACL) guide was inserted through the anteromedial portal to target the center of the avulsion fragment within the posterior capsule (Fig. 3B). The guide was set at a 50° angle with the bullet positioned under the skin at the inferomedial tibial tubercle. A 15 mm incision marked the tunnel exit point, and a 1.5 mm Kirschner wire was drilled along the guide to exit through the center of the avulsion fragment. After the tibial ACL guide was removed, a PCL protector was placed at the tip of the Kirschner wire, and a 3.4 mm cannulated drill was used to create a tibial tunnel, stopping immediately upon exiting the posterior surface of the fragment (Figs 3C and 4A). The cannulated drill bit remained in place for suture passage and the Kirschner wire was removed. Two polydioxanone synthetic absorbable sutures (PDS) II (Ethicon, LLC) were introduced through the cannulated drill to serve as traction sutures, with one end of each PDS II suture exiting through the avulsion fragment and tibial tunnel, and the other end through the anterolateral portal. From the anterior portal, a high-strength suture (Smith & Nephew, USA) was introduced to encircle the PCL (Fig. 3D), with its end connected to one of the PDS II sutures and pulled out of the tibial tunnel. A high-strength suture (Smith & Nephew, USA) attached to a 60 mm looped Endobutton (Smith & Nephew, USA) was passed into the joint between the ACL and PCL. Another PDS II was used to pull the suture and loop the Endobutton through the tibial tunnel, positioning it over the avulsion fragment (Fig. 3E). The direction of the button in the joint is adjusted using two sutures at each end of the button. When applying an anterior draw at 70–90° knee flexion, the loop was tightened until the fracture was fully reduced (Fig. 3F). Finally, A 6 mm interface screw (Smith & Nephew, USA) was inserted into the tibial tunnel to secure both the Endobutton and high-strength sutures (Smith & Nephew, USA) around the PCL. The high-strength suture attached to the loop and suture around the PCL were fixed to the anterior tibial cortex using a tendon fixation anchor (Smith & Nephew, USA) (Fig. 4B). The posterior drawer (e) and Lachmann tests (e) were repeated before closure.

### Postoperative rehabilitation

After surgery, the injured limb was immobilized with a hinged knee brace for 8 weeks. From the first postoperative day, patients were encouraged to perform isometric contractions of the quadriceps and ankle exercises. For the first 3 weeks, the adjustable brace was set at 30 °flexion and the angle was increased by 15 °each week. The brace was removed during flexion and extension. For the initial 6 weeks, walking without touching the ground with the aid of crutches was allowed, and partial-weight walking was allowed from week 6. Walking without crutches was initiated at week 7. Passive range-of-motion exercises began in the second postoperative week, with passive knee flexion increasing to 40–60 °by week 2, and further to 90–120 °between weeks 4 and 8. During the knee flexion exercises, the brace was removed, allowing the patient to sit with the leg falling naturally. The patients were also encouraged to perform active knee extension and flexion exercises. Upon discharge, all patients received a comprehensive rehabilitation plan via WeChat (a communication application). The patient returned to the hospital for follow-up imaging and physical examinations at 6 weeks, 3 months, 6 months, 1 year, and 2 years postoperatively.

At the final follow-up, knee function was assessed using the Lysholm and IKDC subjective scores, whereas the IKDC objective grade was determined through radiographic and physical assessments. Fracture healing was evaluated based on the absence of fracture lines on radiography and/or knee stability observed during the physical examination^[20]^.

## Statistical analysis

Data analysis was conducted using SPSS (version 29.0; SPSS Inc.). The Shapiro-Wilk test was used to determine the normality of continuous variables. Data following a normal or approximately normal distribution were reported as mean ± standard deviation. In this study, Shapiro-Wilk normality tests were conducted on the pre- and postoperative score differences using SPSS. The data followed a normal distribution (*P* > 0.05). Pre- and postoperative Lysholm and IKDC subjective scores were compared using Student’s paired *t* test. To evaluate the reliability of repeated measures, the intraclass correlation coefficient (ICC) was calculated to assess both inter- and intra-rater consistency. An ICC value above 0.75 indicated good reliability; values between 0.50 and 0.75, moderate reliability, and values below 0.50 indicated poor reliability. Nonparametric paired tests were used to compare the preoperative and postoperative IKDC knee examination grades. A *P* value of <0.05 was considered statistically significant.

### Calculation

**Figure 5.**
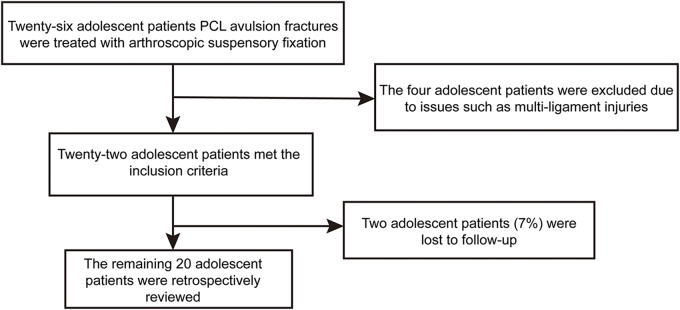
Flowchart outlining the patient selection methodology and criteria. PCL: posterior cruciate ligament.

During the study period, 26 adolescents (age <18 years) with acute (<3 weeks) PCL avulsion fractures were treated using arthroscopic suspension fixation. Among these, 20 patients met the inclusion criteria and completed a minimum of 2 years of follow-up. The patients’ demographic data are summarized in Table 1. Two patients were excluded because the time from injury to treatment exceeding 3 weeks. Another adolescent under 18 years of age was excluded because of closed epiphyseal plates, and one patient with a concurrent anterior cruciate ligament tear was also excluded. Two (7 %) patients were lost to follow-up (Fig. 5).

**Table 1:** Demographic data of patients

Variable	No. of patients or mean (range)
Age, y	15.4 (12–18)
Male/Female	11/9
Mechanism of injury	
*Electric bicycle accident*	10
*Fall from height*	7
*Sports-related injury*	3
Time to surgery, d	8.4 (2–20)
Duration of surgery, min	64.8 (50–90)
Follow-up, mo	29.8 (24–48)

At final follow-up, the mean knee range of motion (ROM) was 136.5 ± 4.12°, allowing patients to resume their normal daily and academic activities. No complications such as neurovascular injury, thrombosis, infection, or implant loosening were reported, including one patient who engaged in high-intensity sports without specific discomfort. The mean Lysholm score increased from 21.55 ± 7.18 preoperatively (range: 8–32) to 95.25 ± 2.17 at the final follow-up (range: 91–99). The mean IKDC subjective score improved from 18.25 ± 5.49 (range: 9–28) to 94.9 ± 2.43 (range: 90–99), and knee ROM improved from a preoperative mean of 39.25 ± 6.71 (range: 27–53) to 136.5 ± 4.12 postoperatively (range: 130–144). Significant improvements were observed in IKDC examination grade (Table 2). The differences between the preoperative and postoperative scores were statistically significant (*P* < 0.05).

**Table 2:** Comparison of statistical data between preoperative and postoperative evaluation

Parameter	Preoperative^a^	Postoperative^a^	95%CI	*P* value
Lysholm score	21.55 ± 7.18(8–32)	95.25 ± 2.17(91–99)	70.17–77.23	<0.001
IKDC subjective score	18.25 ± 5.49(9–28)	94.9 ± 2.43(90–99)	74.24–79.06	<0.001
ROM	39.25 ± 6.71(27–53)	136.5 ± 4.12(130–144)	93.88–100.62	<0.001
IKDC examination grade				0.000
A (normal)	0	10		
B (nearly normal)	0	10		
C (abnormal)	8			
D (severely abnormal)	12			

^a^ Values are presented as mean ± SD (range) or number of patients.

CI, confidence interval, IKDC, International Knee Documentation Committee, ROM, Knee Range of Motion. IKDC examination grade, Grade A (Normal), The knee exhibits full function with no pain, stiffness, or swelling, and the individual is able to engage in all sports and daily activities without restriction, Grade B (Nearly Normal), The knee demonstrates minor symptoms or discomfort, but these do not significantly affect the individual’s ability to perform most daily activities or engage in light to moderate sports, Grade C (Abnormal), The knee shows notable symptoms such as pain, swelling, or instability, which lead to some functional limitations, significantly affecting daily activities or moderate to high-intensity sports, Grade D (Severely Abnormal), The knee is severely affected by symptoms, with substantial limitations in function, including pain, swelling, and instability, which significantly impair daily activities and prevent participation in most sports

The minimal clinically important difference (MCID) in the IKDC subjective score for patients with knee pain was 16.7 points (12 months), and the Patient Acceptable Symptom State (PASS) threshold was set at 75.9 points. The minimal detectable change (MDC) for the Lysholm score was set at 8.9 points, although PASS thresholds for the Lysholm score have not been reported previously^[21]^. All patients in this study achieved both MCID and PASS in the IKDC subjective score at the final follow-up and 100% met the MDC criteria for the Lysholm score. At the last follow-up, the patients were positioned in the supine position with both legs extended and the pelvis level. A tape measure was used to assess the distance from the anterior superior iliac spine to the ipsilateral medial malleolus, and any leg-length discrepancy was calculated. Measurements were performed by two orthopedic surgeons in a double-blind manner, with each surgeon measuring and calculating twice for each patient. The ICC for each parameter was calculated using SPSS 29 to evaluate the inter-surgeon consistency. The ICC values for the individual measurements were 0.89 and 0.88, with an inter-rater reliability ICC of 0.94, and all ICC values exceeded 0.75, indicating high reliability. The average of the two measurements was used as the final result. Measurement data indicated that leg length discrepancies were <1 cm for all patients at the final follow-up, with no observed limping or subjective complaints of leg length discrepancy or limitations in sports or daily activities.

**Figure 6.**
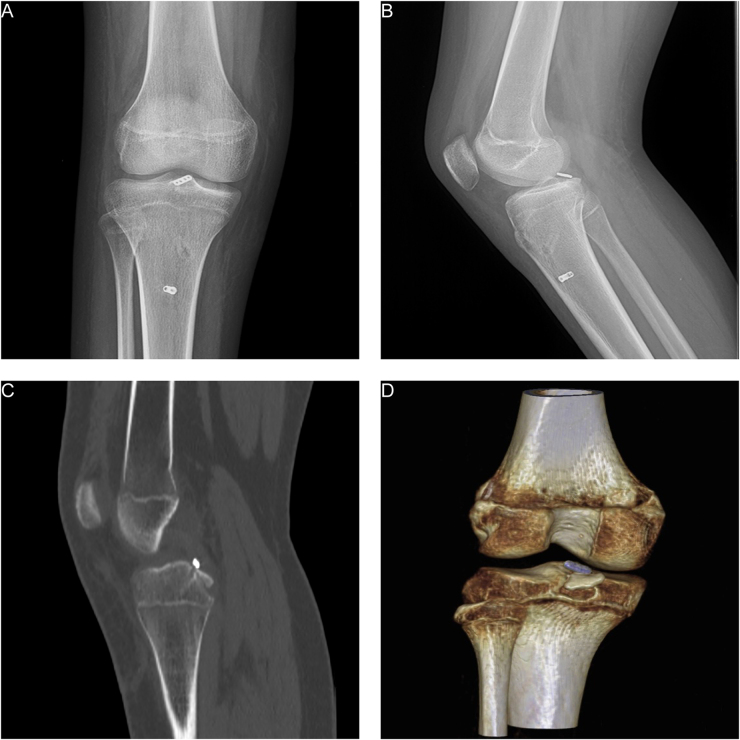
Postoperative imaging evaluation at 3 months. (A) Anteroposterior X-ray confirming proper alignment of the bone. (B) Lateral X-ray revealing stable fixation of the fracture. (C) CT sagittal scan shows successful clinical healing of the fracture. (D) Three-dimensional CT scan illustrating successful clinical union of the avulsion fracture and excellent positioning of the implant.

Postoperative radiographs showed bone union in all cases, including large fragments and comminuted fractures, with no evidence of implant displacement (Fig. 6). At the final follow-up, all the patients reported satisfactory knee function and full recovery.

## Discussion

The principal finding of this study was that arthroscopic treatment of acute adolescent PCL tibial avulsion fractures using suspension fixation devices delivered excellent outcomes in terms of knee joint stability and fracture healing. All cases demonstrate that this technique is both feasible and straightforward to implement in clinical practice.

Debates persist regarding the optimal treatment for acute PCL tibial avulsion fractures in adolescents. Al-Ahaideb pointed out that if the avulsed tibial fragment is not displaced, patients may undergo conservative treatment^[14]^. However, displaced fragments are at risk of failing to heal or causing ligament laxity when treated conservatively. Although the existing literature suggests that adult patients with a displacement exceeding 6.7 mm should undergo surgical treatment^[22]^, no clear surgical guidelines for adolescents have been established.

Presently, many experts recommend surgical fixation of displaced PCL avulsion fragments to restore anatomical alignment and knee stability^[23,24]^. The classical surgical approach involves an open reduction and posterior fixation. However, this method does not allow for the simultaneous management of intra-articular injuries such as meniscal tears or cartilage damage. Arthroscopic techniques, on the other hand, have become a preferred alternative for treating adolescent with PCL tibial avulsion fractures. Studies have shown that arthroscopy offers outcomes comparable to those of open surgery^[25]^. A systematic review revealed that while both approaches were safe and effective, patients treated arthroscopically exhibited higher mean Lysholm scores than those treated with open surgery^[6]^.

In our study, we addressed the issue of small fracture fragments using a high-strength suture to twist the PCL, effectively mitigating the fixation challenges posed by the fragment size. We further used a cortical button (Smith & Nephew, USA) comprising a titanium button and a 60-mm suture loop with a length sufficient to traverse the tibial tunnel. This design minimizes the risk of suture breakage within the bone tunnels. Researchers have validated the biomechanical properties of Endobutton fixation in laboratory settings. Through experiments, such as the Lachman test, cyclic loading test, ultimate failure load test, and stiffness test, they concluded that the structural characteristics of Endobutton fixation are comparable to those of direct screw fixation techniques^[18,26]^.

Our technique significantly minimizes the risk of interference with the growth plates in adolescents. Currently, the most significant concern in adolescent patients with PCL tibial avulsion fractures is whether surgical methods can cause growth disturbances. Lo *et al* performed anterior cruciate ligament reconstruction using a 6 mm tunnel in five adolescent patients. Postoperatively, no leg length discrepancies or angular deformities were seen, and the tibial growth plates closed symmetrically, indicating favorable outcomes^[26]^. Similarly, Nirav K. Pandya successfully treated a 12-year-old girl with an open growth plate using a 4 mm hollow screw and washer. Twelve months after surgery, the patient returned to sports, and physical examination revealed stable and symmetrical knee function^[13]^. Although neither study specifically reported the exact incidence of growth disturbances, the transition from a 6 mm to a 4 mm tunnel highlights the safety advantages of smaller tunnels in terms of postoperative outcomes.

Research has indicated that tunnels smaller than 6 mm do not cause growth disturbances^[27]^, whereas tunnels of 12 mm or larger can lead to growth disturbances^[28]^. Further studies have shown that the risk of growth disturbances is related to the extent of damage to physiological cross-sectional area. Houle JB’s al. reported that growth cessation occurred when 7% of the cross-sectional area of the growth plate was damaged^[29]^. Shea’s three-dimensional study showed that a 6 mm tibial tunnel removed an average of 1.6% of the growth plate area^[30]^. This suggests that the amount of growth plate removed by a 6 mm tunnel is minimal. In contrast, we use a 3.4 mm tunnel, which is only 57% of the diameter of a 6 mm tunnel. This significantly smaller size not only reduces damage to the growth plate but also lowers the risk of growth cessation, offering a higher degree of safety.

At the final follow-up, all patients exhibited leg length discrepancies of less than 1 cm. No observable gait abnormalities were noted during routine walking and running, and no patients reported subjective issues, such as functional impairment or leg length differences. A systematic review of leg length discrepancies highlighted that in Germany, treatment guidelines recommend no intervention for discrepancies <10 mm^[31]^. In growing children, leg length differences greater than 10 mm are considered clinically significant. In growing children, leg length differences greater than 10 mm are considered clinically significant^[32]^.

While suspension fixation does not provide rigid stability for screw fixation and allows for minor movements owing to its elastic nature, our technique has proven to be effective for smaller fracture fragments and even comminuted fractures. The core principle of suspension fixation lies in its elastic properties, which convert the torque generated during knee movement into compressive forces on the avulsed bone fragments^[33]^. This promotes fracture healing while preserving knee-joint stability.

Currently, research on PCL tibial avulsion fractures in adolescents is limited, and our study offers valuable insights into this field. Our findings suggest that the 3.4 mm tunnel technique, in contrast to the traditional larger tunnels (6 mm and 12 mm), not only enhances surgical safety, but also reduces the risk of growth disturbances. Suspension fixation can be adapted for various fracture types, providing a new and safer treatment option for PCL tibial avulsion fractures in adolescents. In future studies, we can optimize the Endobutton fixation material by developing bioactive materials^[34]^, leveraging artificial intelligence to enhance the precision and intelligence of diagnosis and treatment^[35]^, and introducing an individualized treatment model^[36]^ to further advance the research on the treatment of adolescent PCL tibial avulsion fractures.

This study has several limitations. First, as a single-center retrospective study, this design is known to introduce inherent selection bias and may lack scientific rigor for prospective randomized controlled trials. Given the low incidence of PCL tibial avulsion fractures, it was difficult to conduct a prospective study. Second, we did not use quantitative assessment tools, such as the KT-1000 or KT-2000 arthrometers, to evaluate knee stability because these devices were unavailable at our hospital. Consequently, the assessment of knee stability relies primarily on subjective scoring, which can result in an underestimation of instability, particularly in cases of subtle instability. Third, the follow-up duration varied among the patients, which may have introduced bias in the evaluation of postoperative knee function at the final follow-up. Therefore, in future research, we will perform long-term follow-ups until the growth plates of adolescent patients mature to allow for further analysis. Additionally, we plan to collaborate with other centers to conduct a multicenter, prospective, controlled study to provide high-quality evidence.

## Conclusion

The suspension technique under arthroscopic guidance is a simple, effective, and minimally invasive approach for the treatment of tibial avulsion fractures of the PCL in adolescents. This approach delivered remarkable results, ensuring excellent knee-joint stability and reliable fracture healing.

## Data Availability

The data that support the findings of this study are not openly available due to reasons of sensitivity and are available from the corresponding author upon reasonable request.

## Research registration unique identifying number (UIN)

ChiCTR2400086543.
